# The Molecular Mechanisms of OPA1-Mediated Optic Atrophy in *Drosophila* Model and Prospects for Antioxidant Treatment

**DOI:** 10.1371/journal.pgen.0040006

**Published:** 2008-01-11

**Authors:** Will Yarosh, Jessica Monserrate, James Jiayuan Tong, Stephanie Tse, Phung Khanh Le, Kimberly Nguyen, Carrie B Brachmann, Douglas C Wallace, Taosheng Huang

**Affiliations:** 1 Department of Pediatrics, Division of Human Genetics, University of California Irvine, Irvine, California, United States of America; 2 Department of Developmental and Cell Biology, University of California Irvine, Irvine, California, United States of America; 3 Department of Biological Chemistry, University of California Irvine, Irvine, California, United States of America; 4 Center for Molecular and Mitochondrial Medicine and Genetics, University of California Irvine, Irvine, California, United States of America; 5 Department of Pathology, University of California Irvine, Irvine, California, United States of America; Stanford University, United States of America

## Abstract

Mutations in optic atrophy 1 (*OPA1*), a nuclear gene encoding a mitochondrial protein, is the most common cause for autosomal dominant optic atrophy (DOA). The condition is characterized by gradual loss of vision, color vision defects, and temporal optic pallor. To understand the molecular mechanism by which *OPA1* mutations cause optic atrophy and to facilitate the development of an effective therapeutic agent for optic atrophies, we analyzed phenotypes in the developing and adult *Drosophila* eyes produced by mutant *dOpa1* (*CG8479*), a *Drosophila* ortholog of human *OPA1*. Heterozygous mutation of *dOpa1* by a P-element or transposon insertions causes no discernable eye phenotype, whereas the homozygous mutation results in embryonic lethality. Using powerful *Drosophila* genetic techniques, we created eye-specific somatic clones. The somatic homozygous mutation of *dOpa1* in the eyes caused rough (mispatterning) and glossy (decreased lens and pigment deposition) eye phenotypes in adult flies; this phenotype was reversible by precise excision of the inserted P-element. Furthermore, we show the rough eye phenotype is caused by the loss of hexagonal lattice cells in developing eyes, suggesting an increase in lattice cell apoptosis. In adult flies, the *dOpa1* mutation caused an increase in reactive oxygen species (ROS) production as well as mitochondrial fragmentation associated with loss and damage of the cone and pigment cells. We show that superoxide dismutase 1 (SOD1), Vitamin E, and genetically overexpressed human SOD1 (hSOD1) is able to reverse the glossy eye phenotype of *dOPA1* mutant large clones, further suggesting that ROS play an important role in cone and pigment cell death. Our results show *dOpa1* mutations cause cell loss by two distinct pathogenic pathways. This study provides novel insights into the pathogenesis of optic atrophy and demonstrates the promise of antioxidants as therapeutic agents for this condition.

## Author Summary

Optic atrophies are a group of neurodegenerative disorders characterized by a gradual loss of vision, color vision defects, and temporal optic pallor. Autosomal dominant optic atrophy (DOA), a type of optic atrophy, contributes to a large portion of optic atrophy cases. Mutations of the optic atrophy 1 (*OPA1*) gene are responsible for this condition. Here we describe mutant *Drosophila* that contain insertions in the *Drosophila* OPA1 ortholog, *dOpa1*. Heterozygous mutation causes no discernable eye phenotype, and homozygous mutation results in embryonic lethality. Using the powerful *Drosophila* genetic techniques, we created eye-specific mutants, giving rise to cells with two mutant copies of *dOpa1* only in the *Drosophila* eye, and found that these eyes were rough (mispatterned) and glossy (decreased lens and pigment deposition). We found that these phenotypes were associated with fragmented mitochondria and were caused by elevated reactive oxygen species. The administration of antioxidants could ameliorate the phenotypes caused by mutation of *dOpa1*, offering new insight into treatment of this disease.

## Introduction

Autosomal dominant optic atrophy (DOA) is the most common hereditary optic atrophy with an incidence rate as high as 1:10,000–1:50,000 [1,2]. It is characterized by central vision loss, color vision abnormalities [1,3–5], and degeneration of the retinal ganglion cells [6]. The onset of DOA symptoms typically occur in the first decade of life and visual loss is progressive, bilateral and irreversible once cell death has occurred [3]. There is marked intra- and inter-familial phenotypic variability [5,7]. Optic atrophy may associate with hearing loss, apoptosis, and ophthalmoplegia [8]. Some families have been described with sex-influenced DOA phenotypes [9–11].

The majority of DOA is caused by mutations in the optic atrophy 1 (*OPA1*) gene [7,12–14]. *OPA1* is a nuclear gene which encodes a mitochondrial protein [12]. OPA1 consists of an N-terminal mitochondrial target signal, a transmembrane domain, a presenilin-associated rhomboid-like protease recognition site, and a dynamin-like domain with GTP binding activity. OPA1 is expressed ubiquitously [13,15] and functions in mitochondrial fusion [16–22], ATP production [23], and cytochrome-c mediated apoptosis [24]. Mitochondrial diseases are associated with mitochondrial fragmentation due to the fast proteolytic processing of the OPA1 protein. Overexpression of OPA1 can prevent such fragmentation [18]. These observations suggest that OPA1 plays an important role in mitochondrial function.

Although studies have contributed to our current understanding of OPA1 in terms of function and its relationship to optic atrophy, the pathogenesis of optic atrophy remains poorly understood. The major goal of this study is to establish an animal model for effective *in vivo* OPA1 studies for pathogenesis and therapeutic development. Previous attempts to create a mouse model of *OPA1* revealed that homozygous *Opa1* mutant mice are embryonically lethal, while heterozygous animals showed no phenotype until a later age [25,26]. A *Drosophila* model of optic atrophy would offer several advantages: there is excellent homology between *Drosophila* genes and many human disease loci; it has a short lifespan; its eye development is well-studied; its genome has been sequenced; a wide variety of mutants and gene manipulation systems are available; and, for optic atrophy studies, its eye structure is well-defined and the phenotype is easily visualized [27]. During *Drosophila* eye development, lattice cell apoptosis is highly regulated, with one-third of the lattice cells being eliminated by apoptosis.

Here, we established a *Drosophila* model where the *Drosophila* ortholog of *OPA1*, *CG8479* (*dOpa1*) [28] is inactivated by two different insertions. Furthermore, we were able to produce somatic homozygous *dOpa1*-deficient cells in *Drosophila* eyes. Functions of OPA1 in the eye and pathogenesis of optic atrophy were also addressed.

## Results

### *Drosophila Opa1* Is an Ortholog of Human *OPA1*

To identify the *Drosophila* ortholog of human *OPA1*, we performed BLAST searches with the human *OPA1* cDNA and amino acid sequences. *CG8479* (GenBank [http://www.ncbi.nlm.nih.gov/Genbank], protein accession number: AAF58275) was identified as a *Drosophila* ortholog of *hOPA1* (E = 0.0). To further characterize *dOpa1*, we used multiple sequence comparison by log expectation (MUSCLE) algorithm to perform amino acid sequence alignments of human OPA1 (hOPA1), mouse Opa1 (mOpa1), CG8479 (dOPA1), and Yeast Mgm1 (Figure S1). In all four organisms, the GTPase domain was the most conserved region and the basic domain was the least conserved region. The amino acid sequence of mOPA1 shared 96% similarity with hOPA1. When regions of these proteins were compared, the similarity percentages were 86.5% for the basic domain, 99.6% for the GTPase domain, and 98% for the dynamin central region. dOpa1 shared 51.2% similarity to hOpa1 amino acid sequence, a higher score than the well-studied yeast protein Mgm1. At the domain level, the similarity scores were 24.5% for the basic domain, 72% for the GTPase domain, and 53.5% for the dynamin central region. The high amino acid conservation of the GTPase domain and the dynamin regions between hOPA1 and dOpa1 suggests that dOpa1 is the *Drosophila* ortholog of hOPA1.

**Figure 1:**
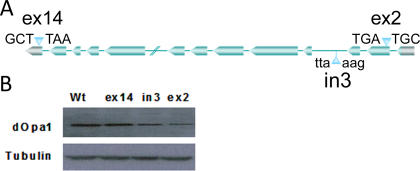
Insertion in *dOPA1* Can Disrupt dOPA1 Expression The location of each within *dOpa1* and the nucleotide sequences flanking the insertion site in the *Drosophila* lines used in this study (A). Western blot analysis of several *Drosophila* lines containing insertions near or within *dOpa1*. (B) shows *dOpa1* levels in adult *Drosophila* wild-type *dOpa1* (*dOpa1* ^+/+^), *Drosophila* with a transposon insertion in exon 14 (noncoding region) (*dOpa1* ^+/ex14^), intron 3 (*dOpa1* ^+/in3^), and exon 2(*dOpa1* ^+/ex14^). Tubulin was used as a loading control.

Three *Drosophila* lines with P-element/transposon insertions in the *dOpa1* gene were used in this study. The insertions in the coding sequence of 2^nd^ exon, in the 3^rd^ intron, and in the noncoding region of the 14^th^ exon of the *dOpa1* gene were designated as *dOpa1* ^+/ex2^, *dOpa1* ^+/in3^, and *dOpa1* ^ex14/ex14^, respectively (Figure 1A; http://www.flybase.org). To determine if these insertions disrupted *dOpa1* expression, we examined dOpa1 protein levels in these lines. Western blot analysis with a mouse polyclonal antibody against OPA1 showed that wild-type and *dOpa1* ^ex14/ex14^ flies had similar levels of dOpa1 (Figure 1B), indicating that the insertion in exon 14 had no effect on *dOpa1* expression. In contrast, the protein levels in *dOpa1* ^+/ex2^ and *dOpa1* ^+/in3^ were decreased. This indicated that the P-element insertion in exon 2 (*dOpa1* ^+/ex2^) and transposon insertion in intron 3 (*dOpa1* ^+/in3^) disrupted *dOpa1* expression. Since insertion in non-coding exon 14 had no effect on the dOpa1 protein level, *dOpa1* ^+/ex14^ served as a control.

### Somatic Homozygous Mutations in *dOpa1* Resulted in a Rough and Glossy Eye Phenotype in Adult *Drosophila* Flies

**Figure 2:**
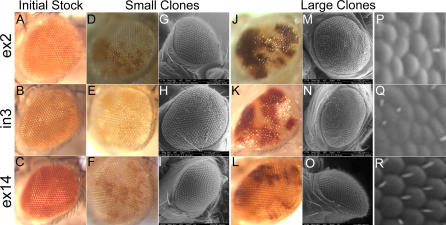
Homozygous Mutation of *dOpa1* Results in a Rough and Glossy Phenotype in the Somatic Clones of the Adult *Drosophila* Eye (A–C) Bright field microscopy images of the adult eyes of the original heterozygous insertion lines are shown in (A), (B), and (C) for *dOpa1* ^+/ex2^, *dOpa1* ^+/in3^, and *dOpa1* ^+/ex14^, respectively. None of these stocks contained any gross eye phenotype. The mosaic-eyed flies produced by the F2 cross (small clones) contain *dOpa1* ^+/+^, *dOpa1* ^+/−^, or *dOpa1* ^−/−^ cell types, which are white, light orange, and dark orange, respectively. (D–I) (D), (E), and (F) are bright field images and (G), (H), and (I) are SEM micrographs of the small clones *dOpa1* ^ex2^, *dOpa1* ^in3^, and *dOpa1* ^ex14^ mutations, respectively. A weak rough phenotype with low penetrance was observed in the small clones of the *dOPA1* ^ex2^ and *dOPA1* ^in3^ mutations, but not the *dOPA1* ^ex14^ mutation. (J–R) (J), (K), and (L) are bright field images; (M), (N), and (O) are SEM micrographs; and (P,Q,R) are 10× digital zooms of areas of interest in the large clones of the *dOpa1* ^ex2^, *dOpa1* ^in3^, and *dOpa1* ^ex14^ mutations, respectively. Glossy and rough phenotypes were observed with 100% penetrance in the large clones of the *dOPA1* ^ex2^ and *dOPA1* ^in3^ mutations, but not the *dOPA1* ^ex14^ mutation.

After we identified the *Drosophila* ortholog of *OPA1*, *dOpa1,* and confirmed that the insertions in the coding sequence of the 2^nd^ exon and the 3^rd^ intron disrupted *dOpa1* expression, we proceeded to test if loss of *dOpa1* would produce an eye phenotype. Eye phenotypes of *dOpa1* ^+/ex2^, *dOpa1* ^+/in3^, and *dOpa1* ^+/ex14^ flies were examined by bright field microscopy (Figure 2A–2C). No gross eye phenotypes were observed, suggesting that haplo-insufficiency did not produce an observable phenotype in the *Drosophila* eye.

We then crossed *Drosophila dOpa1^+/ex2^*, *dOpa1^+/in3^*, and *dOpa1^+/ex14^*. No *dOpa1^ex2/ex2^* or *dOpa1^in3/in3^* flies were obtained, whereas *dOpa1* ^ex14/ex14^ flies were produced and appeared normal. Therefore, homozygous *dOpa1^ex2^* and *dOpa1^in3^* appear to be lethal, supporting the conclusion that the insertions in exon 2 (*dOpa1* ^+/ex2^) and intron 3 (*dOpa1* ^+/in3^) disrupted *dOpa1* expression. To generate *Drosophila* with homozygous *dOpa1^in3/in3^* mutant ommatidia without being impeded by the embryonic lethality of the homozygous mutant, we generated homozygous mutant ommatidia from heterozygous *Drosophila* by using somatic mutagenesis to eliminate the *dOpa1^+^* allele in certain eye cells.

The crosses with the *dOpa1* ^+/−^ *Drosophila* (Figure S2A) resulted in mosaic-eyed flies by the F2 cross (small clones). The mutant ommatidia contained three different cell types of either *dOpa1* ^+/+^, *dOpa1* ^+/−^, or *dOpa1* ^−/−^. The different cell types could be identified by eye color (white, light red, and red, respectively), which corresponded to an increased copy number of the mini-white gene in the insertions. Small clone mosaics were generated for *dOpa1* ^ex2^, *dOpa1* ^in3^, and *dOpa1* ^ex14^, and later characterized by bright field and scanning electron microscopy (Figure 2). The homozygous mutant clones (*dOpa1* ^ex2/ex2^ and *dOpa1* ^in3/in3^) had a slightly rough eye phenotype with low penetrance (<5%–10%) compared with the respective parental (Figure 2D–2H) and control *dOpa1* ^ex14/ex14^ (Figure 2F and 2I) stocks.

To analyze the phenotype of the somatic homozygous *dOpa1* mutants with greater sensitivity, we generated “large somatic clones” in the *Drosophila* eye (Figure 2J–2R) using a mutation in the *Minute* gene located on the same chromosomal arm as *dOPA1*. Homozygous mutations of the *Minute* gene are lethal, therefore eliminating *Minute* ^−/−^ *dOpa1* ^+/+^ cells. As a result, generation of large somatic clones produced only two cell types: *dOpa1* ^+/−^ *Minute* ^+/−^ and *dOpa1* ^−/−^ *Minute* ^+/+^ [29]. We successfully generated large clone mosaics for insertions in exon 2 (Figure 2J, 2M, and 2P), intron 3 (Figure 2K, 2N, and 2Q), and exon 14 (Figure 2L, 2O, and 2R). In *dOpa1* ^ex2/ex2^ and *dOpa1* ^in3/in3^, but not the *dOpa1* ^ex14/ex14^ clones, had a robust rough/glossy phenotype. In addition, *dOpa1* ^ex2/ex2^ and *dOpa1* ^in3/in3^, but not *dOpa1* ^ex14/ex14^, clones exhibited tissue necrosis with variable onset and penetrance (Figure S3). The large clones of the *Minute* stock have a darker eye color. Thus, cells containing one copy of the *Minute* mutation were characterized by a very deep red pigmentation. Light color cells represented *dOpa1* ^−/−^ cells. The phenotypes of *dOpa1* exon 2, intron 3, and exon 14 large clone mosaic eyes were scored (*n* > 60; Figure S2B). Almost all of the *dOpa1* exon 2 and intron 3 large clones exhibited a severe rough/glossy phenotype, while all of the *dOpa1* exon 14 large clones appeared normal.

### The *dOpa1* Mutation Is Genetically Reversible Via P-Element Excision

**Figure 3:**
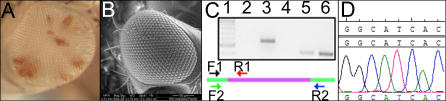
Reversal of the *dOpa1* Large Clone Eye Phenotype by P-Element Excision The phenotypes present in large clones of the *dOpa1* ^ex2^ mutation were reversed, as documented by bright field microscopy (A) and SEM (B), by excision of the P-element. The excision of the P-element insertion was verified by PCR (C). Lane 1 contains 1 μg of the 100 base pair ladder (NEB) for reference. Lane 2 is a negative reagent control without template DNA. Lane 3 contains DNA from *dOpa1* ^+/ex2^ amplified with the F1 primer, which anneals in the exon 2 flanking the P-element insertion and the R1 primer annealing inside the P-element. Lane 4 contains DNA from excised flies amplified with the F1 and R1 primers. Lane 5 contains DNA from *dOpa1* ^+/ex2^ and is amplified using F1 and R2. Lane 6 contains DNA excised from flies amplified with F1 and R2. A schematic representation of the annealing sites of the primer sets used in (C). We further verified the P-element excision by sequencing of the PCR product generated in lane 6 (D) and comparing with the known wild-type (wt) sequence.

To confirm that the phenotype of the somatic clones was the result of disrupting *dOpa1* expression by the P-element insertion, we excised the P-element by crossing the stocks with KiΔ2–3 stocks. KiΔ2–3 stocks express a transposase that excises the P-elements (Figure S4). Excision of the P-element in *dOpa1* ^ex2/ex2^ resulted in a complete reversal of the rough/glossy phenotype in the large clones (Figure 3A and 3B). The P-element excision was confirmed by PCR using primers flanking the P-element insertion site. As expected after complete excision, the PCR amplification produced a 413 base pair fragment (Figure 3C, Lane 6). The precision of the excision was verified by DNA sequencing (Figure 3D). These results show that the rough glossy eye phenotype observed in our *dOpa1* somatic clones was indeed caused by a P-element insertion in the *dOpa1* gene, which can be genetically reversed.

### Homozygous Mutation of *dopa1* Causes Interommatidial Cell Death

All ommatidial units are uniformly patterned by the specific placement of interommatidial cells (IOC), also called lattice cells. It has been shown that apoptosis helps achieve the final pattern through removal of surplus IOC during development. The core of an ommatidium consists of four cone cells and two primary pigment cells, which are surrounded by IOC. The rough phenotypes observed in the adult *Drosophila* eye of the *dOpa1^−/−^* somatic clones suggest that cells within the ommatidium fail to develop and pattern properly, presumably due to cells failing to regulate IOC apoptosis properly. The glossy phenotype is indicative of a failure to deposit lens and pigment material by the cone and pigment cells.

Large *dOpa1* somatic clones were generated with the *Minute* gene mutation and GFP under control of a ubiquitous promoter (ubi), both of which are distal to the flippase recombination target sequence (FRT). As a result, the *dOpa1* ^in3/in3^ *Minute* ^−/−^ *ubi-GFP* ^−/−^ (GFP negative) clones can be distinguished from the *dOpa1* ^+/in3^ *Minute* ^+/−^ *ubi-GFP* ^+/−^ (GFP positive) clones [30]. To visualize the final, post-apoptotic ommatidial pattern during pupal development, pupae were isolated and dissected 42h after pupal formation (APF). Eye discs were stained using anti-armadillo antibody and Hoechst (Figures S5A and S5B and 4). GFP positive ommatidial units (*dOpa1* ^+/in3^ *Minute* ^+/−^ *ubi-GFP* ^+/−^) had similar numbers of IOCs to those reported for wild-type eyes, but GFP-negative ommatidial units (*dOpa1* ^in3/in3^ *Minute* ^−/−^ *ubi-GFP* ^−/−^) had a significantly reduced number of IOCs (*p* = 5.05 × 10^−8^) associated with a high degree of mispatterning (Figure S5C). Cone and pigment cells of both genotypes were normal at this stage. The decreased number of IOCs in these clones indicates that mutation of *dOpa1* may increase IOC cell death prior to 42h APF, and the reduction of IOCs results in the rough phenotype (mispatterning).

Previously, it was reported that mutations in OPA1 caused a decrease in ATP production [23] and an increase in cytochrome-c release [16,19,24,31–34]. Reduced ATP production caused by inhibited oxidative phosphorylation (OXPHOS) would, in turn, be expected to increase mitochondrial reactive oxygen species (ROS). Chemically increased ROS can damage macromolecules and cellular membranes and increase apoptosis rates. To determine if increased ROS production resulted in increased IOC cell death in the *dOpa1* mutant, pupal retinae were stained for ROS production using dihydroethidium at different stages of development [35]. We did not observe dihydroethidium positive nuclei in any of the *dOpa1* ^in3/in3^ clones (data not shown). This data suggests that the mutation of *dOpa1* did not cause a significant increase in ROS production in the pupal retina prior to 42h APF.

**Figure 4:**
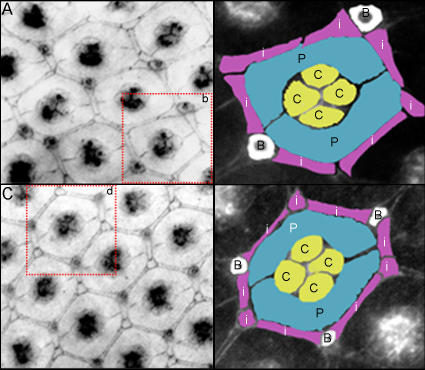
Homozygous Mutation of *dOpa1* Causes a Loss of Interommatidial Cells Pupae were staged 42 h after pupae formation (APF), and large clone mosaics with GFP expression in the eye imaginal disk were collected and stained with Hoechst and for Armadillo. Eye discs were then analyzed and regions of *dOpa1* ^in3/in3^ and/or *dOpa1* ^+/in4^ ommatidial units were photographed and presented (A,C), using only channels with Armadillo signal. (A) illustrates a region of the *dOpa1* ^in3/in3^ ommatidial units. A red box marked with a b is used to indicate the ommatidial unit illustrated in (B). The different cell types are highlighted (B). The cone cells, c, are illustrated in yellow, the pigment cells, p, in blue, the IOCs in purple, and the bristle cells, b, in white. (C) shows the ommatidial units of a *dOpa1* ^+/in3^ Minute^+/−^ ubi-GFP^+/−^ clonal region. A red box marked with a d indicates the ommatidial unit is shown in (D). No cell types are missing, and no disorganization is present. Cell types are represented as in (B).

### Ommatidial Units of Adult Somatic Homozygous *dOpa1* Mutant Flies Are Morphologically Perturbed

**Figure 5:**
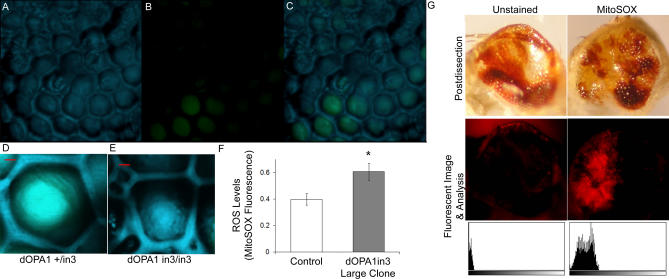
Homozygous *dOpa1* Mutations Produce Morphologically Perturbed Ommatidial Units Confocal microscopy analysis of the eyes of anesthetized adult *dOpa1* ^in3/in3^ large clones. A 3D reconstruction of a large clone with *dOpa1* ^+/in3^ and *dOpa1* ^in3/in3^ ommatidia was generated from reflectance (A), GFP fluorescence (B), and merged signals (C). *dOpa1* ^+/in3^ and *dOpa1* ^in3/in3^ ommatidial units from (C) were magnified and are shown in (D) and (E), respectively. Note the abnormal morphological features of the cone cells in the *dOpa1* ^in3/in3^ ommatidium. The red bar equals 2 microns. (F) shows that *dOpa1* ^in3^ mutation causes higher ROS levels than control eyes. The MitoSOX fluorescent signals were measured in tissue homogenates from dissected control eyes (wild-type) and *dOpa1* ^in3^ large clone eyes. The data represent the mean ± standard deviation of three experiments, using 10-d-old flies and a total of 40 flies per genotype, * < 0.05. ROS level indicated as MitoSOX fluorescence intensity normalized to microgram of eye tissue homogenate. dOpa1^in3^ large clones exhibit relatively higher levels of ROS in dOPA1^−/−^ cells than dOpa1^+/−^ mutant cells. Adult *dOpa1* mutant large clone eyes were promptly dissected, stained, and imaged using MitoSOX in HBSS (G). MitoSOX fluorescent signal histogram plots (bottom row) of fluorescent images (middle row) were generated using jImage (values 0–256, left to right); corresponding light microscope images (top row) illustrate the eye after dissection and MitoSOX staining.

The glossy eye phenotype observed in the *dOpa1* ^in3^ large clones suggests the cone and pigment cells did not secrete and deposit pigment properly [36]. A lens-depleted glossy eye phenotype was previously reported and had a defect in cone and pigment cell specification or proliferation [36,37]. At 42h APF, the pigmented cone cells appeared to be normal in morphology and number. To test if the glossy phenotype in our somatically generated *dOpa1* ^−/−^ ommatidial units was caused by cone and pigment cell death later on, we performed confocal microscopy on the adult *dOpa1* ^in3^ large clones. This technique enabled the visualization of heterozygous (GFP-positive) and homozygous (GFP-negative) mutant ommatidial units, and the dissection of the ommatidial structures at a thickness of 2 microns. Using this approach, it was possible to reconstruct the three-dimensional structure of the *Drosophila* eye. The ommatidial structure of the *dOpa1* ^−/−^ (GFP negative) eye was disorganized (Figure 5; Videos S1–S3), whereas the *dOpa1* ^+/−^ ommatidia (GFP positive) were largely normal. The detailed structure of *dOpa1* ^+/−^ ommatidia (GFP positive) revealed that they were structurally normal; the lattice cells were arranged in hexagonal shapes with cone and pigment cells centralized in the ommatidia (Figure 5; Videos S1–S3). In contrast, *dOpa1^−/−^* (GFP negative) ommatidia showed a loss of lattice cells (consistent with results in Figure 3), the cone cells were condensed, and exhibited severe cell surface defects. The space between cone and lattice cells was also dramatically increased, which is consistent with cone and/or pigment cell death. Our results suggest that the glossy phenotype in somatic mutant eyes was caused by damage or death of cone and pigment cells occurring after 42 h APF, which results in a decreased secretion of lens deposition.

To test if increased ROS levels could cause cone and pigment cell death in adult *dOpa1^−/−^* large clone eye tissues, we measured the ROS levels in the *dOpa1* ^in3/in3^ clones using MitoSOX (Invitrogen, M36008). *dOpa1* ^in3/in3^ large clones exhibited increased ROS levels in the adult *Drosophila* eyes (Figure 5). Thus, the increase in ROS may be correlated with an increase in pigment and cone cell death, which is consistent with the decrease in production of lens material and glossy eye phenotypes. Using MitoSOX staining, we also show that *dOpa1* ^in3/in3^ large clones exhibit higher levels of ROS in *dOpa1^−/−^* cells compared to *dOpa1* ^+/−^ cells (Figure 5). In this experiment, adult *dOpa1* mutant large clone eyes were quickly dissected and stained using MitoSOX (2.5mM) in HBSS. MitoSOX staining on live *dOPA* ^+/−^ large clones eyes were performed on adult *Drosophila* as described recently [38]. As shown in Figure 5, a significantly higher level of MitoSOX fluorescence was detected in *dOpa1* ^−/−^ cells compared to *dOpa1* ^+/−^ cells. The specificity of MitoSOX for superoxide was tested by performing MitoSOX stains in the presence of both SOD-1 (1000 units/ml) and Vitamin E (200 μg/mL); both dramatically reduced MitoSOX signal levels (data not shown).

### The *dOpa1* Mutation Affects Mitochondrial Morphology and Tissue Integrity

**Figure 6:**
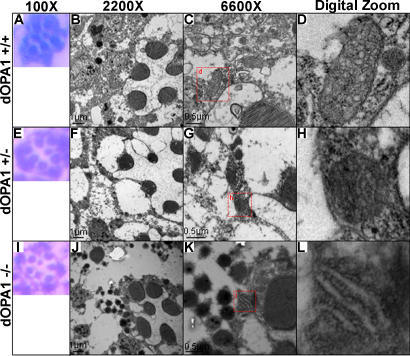
The *dOpa1* Mutation Affects Mitochondrial Morphology and Tissue Integrity Whole flies were sectioned and analyzed by hematoxylin and eosin (H&E) staining and standard microscopy (A), (E), (I) or TEM (B–D), (F–H), (J–L). (C), (G), and (H) contain red boxes with d, g, and i in them, respectively, indicating the region digitally zoomed in (D), (H), and (I). As shown in (A–C), there are no differences in the number of rhabdomeres among *dOpa1* ^+/+^ (A), *dOpa1* ^+/in3^ (E), and *dOpa1* ^in3/in3^ (I) ommatidial units (*n* > 40). Note: TEM analysis of similar sections revealed a significant difference in mitochondrial morphology along with abnormalities in the cells surrounding the rhabdomeres. *dOpa1* ^+/+^ ommatidial units contained many mitochondria (B–D). *dOpa1* ^+/in3^ ommatidial units contained fragmented mitochondria (F–H). *dOpa1* ^in3/in3^ ommatidial units had few mitochondria (J–L), which all had very perturbed morphology. Severe tissue damage is visible in regions within *dOpa1* ^in3/in3^ ommatidial units. *dOpa1* ^in3/in3^ ommatidial units were classified based on the morphology (number of sides) of the ommatidial units; we had previously observed that *dOpa1* ^in3/in3^ ommatidial units adjacent to other *dOpa1* ^in3/in3^ ommatidial units had four rather than normal six sides.

Previous studies have shown that OPA1 mutations induce mitochondrial fragmentation [13]. To determine if loss of *dOpa1* had similar effects on mitochondrial integrity in the *Drosophila* eye, we examined the morphology of mitochondria in wild-type and mutant large clone (*dOPA1^−/−^*) ommatidia. No difference was found in rhombomere structure (Figure 6A, 6E, and 6I), which suggests that the photoreceptor cells were not affected by the *dOpa1* mutation. In the homozygous mutants, mitochondria were scarce and significantly dysmorphic (Figure 6J and 6K). These results suggest that *dOpa1* is important for mitochondrial morphology and that mutation of *dOpa1* may be associated with increased ROS production, leading to cone and pigment cell death.

### The Rough Glossy Phenotype Can Be Partially Reduced by Antioxidant SOD-1, Vitamin E, and Genetically Expressed Human SOD1

**Figure 7:**
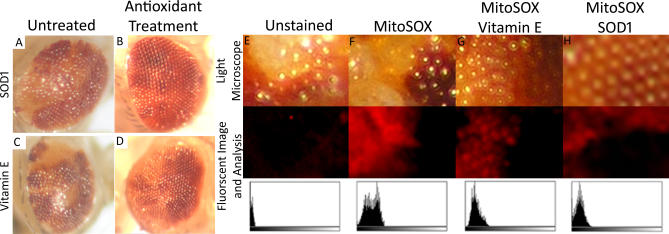
Partial Reversal of the Rough, Glossy Phenotype of *dOpa1* Mutants by the Antioxidants SOD-1 and Vitamin E *dOpa1^in3^* large clones were either untreated, (A) and (C), or treated with 1,000 units/ml SOD-1 (B) or 20 μg/ml vitamin E (D). Antioxidant treatment resulted in partial reversal of the rough eye phenotype. *dOpa1* mutant large clones that received no treatment (E, F), vitamin E (G), or SOD-1 (H) were dissected and stained with MitoSOX (F–H) to visualize ROS levels. *dOpa1* mutant large clones treated with antioxidants displayed lower levels of MitoSOX fluorescence in *dOpa1^−/−^* cells compared to untreated samples. MitoSOX fluorescent signal histogram plots (bottom row) of fluorescent images (middle row) were generated using JImage (Values 0–256, left to right); corresponding light microscope images (top row) illustrate the eye after dissection and MitoSOX staining.

**Figure 8:**
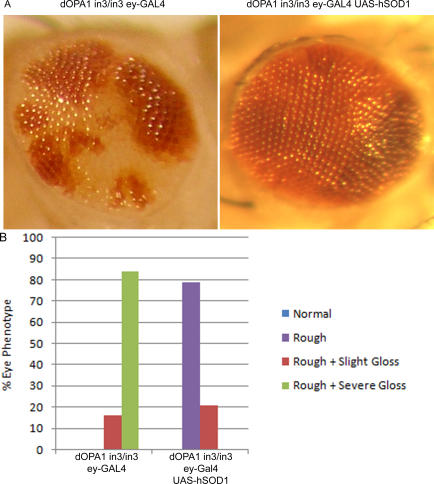
Overexpression of *hSOD1* Reverses the Glossy Eye Phenotype of *dOPA1* Mutant Large Clones Bright field microscopy images of the adult eyes with (A) (left), and without (A) (right) *hSOD1* gene. Adult *dOPA1* mutant large clones were scored for the severity of a glossy eye phenotype (B). Eyes that contained more than 20% glossy ommatidial units in *dOPA1* homozygous mutant (or wild-type chromosomal arm equivalent) were given a score of severe gloss. Eyes of an intermediate phenotype (less than 20% glossy ommatidial units) were given a score of slight gloss. dOPA1^in3^ *UAS-hSOD1*; *ey-Gal4* large clones were generated using *UAS-hSOD1* transgenic flies, kindly provided by R. Bodmer (35) (Figure S6), *eyeless*-*GAL4* (*ey-Gal4*) transgenic flies (Bloomington). Presence of dOPA1^in3^, *UAS-hSOD1*, and *ey-Gal4* were all verified by PCR with primers as described in Materials and Methods.

Our observation that ROS production was increased in the homozygous *dOpa1^−/−^* mutant ommatidia and was associated with cone and pigment cell death suggested that antioxidants might ameliorate the glossy eye phenotype. In previous studies, feeding *Drosophila* with SOD-1 and vitamin E have been shown to inhibit cell death in the Parkinson's disease *Drosophila* model [39]. To test the effects of antioxidants on *Drosophila* eye phenotype, we added SOD-1 and vitamin E to the *Drosophila* food. Ingestion of SOD-1 (1000 units/ml) and vitamin E (20 μg/ml) partially rescued the glossy eye phenotype in the *dOpa1* ^in3/in3^ mutant ommatidum (Figure 7A–7E). Furthermore, MitoSOX (2.5 mM) staining of dissected *Drosophila* eyes showed treatment of antioxidants reduced the ROS level in *dOpa1* ^in3/in3^ cells (Figure 7G and 7H) compared to those which were untreated (Figure 7F). A significant (*p* < 0.05) reduction of ROS levels in *dOpa1^in3^* large clone eye tissue was also found in the homogenates of antioxidant-treated samples (data not shown). In order to further demonstrate that the glossy eye phenotype of *dOPA1* mutant large clones arises as a result of excessive ROS generation by the mitochondria, we genetically tested if overexpression of human superoxide dismutase 1 (hSOD1, GenBank Accession Number NM_000454) is able to reverse the glossy eye phenotype of *dOPA1* mutant large clones. As shown in Figure S6, the overexpression of hSOD1 in the *Drosophila* eye was achieved using a UAS/Gal4 system as previously described [39]. Figure 8A shows that expression of hSOD1 in the *Drosophila* eye resulted in a significant reduction of glossy eye phenotypes of *dOPA1* mutant large clones (Right) in comparison to *dOPA1* mutant large clone controls without the UAS-*hSOD1* transgene (Left). The reversal of the rough eye phenotype of *dOPA1* mutant large clones was not observed. In order to quantify the glossy eye phenotypes of our large clones, the phenotypes of *dOPA1* mutant large clone eyes were scored based on the eye phenotype (*n* > 40). As shown in Figure 8B, overexpression of hSOD1 in the *Drosophila* eye resulted in dramatic reduction of the glossy eye phenotype of *dOPA1* mutant large clones. The use of genetic methods to reverse the glossy eye phenotype of *dOPA1* mutant large clones through the expression of hSOD1 indicates that this phenotype arises as a result of excessive ROS generation by the mitochondria and further supports a role for ROS in the pathogenesis of optic atrophy, suggesting that antioxidants may be beneficial in ameliorating these symptoms.

## Discussion

Here, we report that the somatic mutation of the *Drosophila* OPA1 ortholog, *dOpa1*, results in a rough/glossy eye phenotype. It is very interesting that mutation of *OPA1* in humans causes an autosomal dominant condition. When analyzing eye phenotype of the *Drosophila* eye we found that *Drosophila* heterozygous for mutations in *dOPA1* did not appear to have a gross phenotype. There are many possible explanations for this observation, some of which include the evolutionary differences between *OPA1* in humans and its orthologs. However*,* the grossly normal eye structure in heterozygous mutant *Drosophila* does not imply normal function. It is still possible that the eyes are dysfunctional even though the eye structure is unaffected. We are in the process to analyze the heterozygous mutants for cardiac, skeletal muscle and photoreceptor function.

In the developing *Drosophila* eye, the imaginal disc undergoes two phases of proliferation. First, all cells divide rapidly and asynchronously without regulation [40]. Then, the morphogenic furrow sweeps across the eye disc to pattern the cells. Subsequently, the cells differentiate into photoreceptor cells or undergo another round of division, which produces cone and pigment cells. During this process, the photoreceptor cells are specified first, they then retract and recruit four cone cells, which in turn recruit two pigment cells [41]. The cells remaining from the first phase of proliferation form ommatidial lattice cells [27]. These include the secondary, tertiary, and bristle cells that separate adjacent ommatidia, forming the precise hexagonal patterns of the adult *Drosophila* eye. After cell expansion, excess cells are eliminated by apoptosis [42]. Our results show that the rough/glossy-eye phenotype of the *dOpa1* mutants is associated with lattice and cone/pigment cell death, and that death of lattice cells and cone/pigment cells occur at different developmental stages. In developing *Drosophila* eye, the loss of *dOpa1* causes a significant reduction in the number of lattice cells. However, the cone and pigment cells are structurally normal at 42 h APF stage, but later damage or death of cone and pigment cells results in decrease secretion of lens materials.

The underlying mechanism causing increased lattice cell death due to dOpa1 loss is still unknown. Previous studies showed that lattice cells respond to signals from cone and pigment cells [43]. Our results show that loss of *dOpa1* causes a significant reduction in the number of lattice cells in the presence of normal cone and pigment cells, indicating that death of lattice cells is not due to early defects in differentiation of retinal cell types. It has been shown that OPA1 reduces mitochondrial cytochrome-c release following cristae remodeling in the presence of activators of the mitochondrial apoptosis pathway [16]. Furthermore, electron microscopy and electron tomography studies suggest that OPA1 sequesters cytochrome-c within mitochondrial cristae by narrowing cristae junctions, thereby inhibiting the redistribution of cytochrome-c to the inner membrane space [16]. However, the role of cytochrome-c in apoptosis in *Drosophila* has only been recently established. Mendes and colleagues showed that *cyt c-d* plays an important role in *Drosophila* eye apoptosis; and mutation of *cyt c-d* causes an increase in the number of interommatidial cells and perimeter cells [44]. Thus we hypothesize that lattice cell death is the result of a cell autonomous death signal from mitochondria.

Our results show that cone and pigment cell death was correlated with increased ROS levels and mitochondrial fragmentation. This implies that the increased ROS production induces cell death, which is further supported by the reversal of these effects and the rescuing of the glossy eye phenotype by antioxidants. Increased ROS production can trigger apoptosis to eliminate deleterious cells [45], but the exact link between oxidative stress and specific apoptosis pathways remains elusive. Transient fluctuations in ROS levels may serve as an important regulator of mitochondrial respiration, but high and sustained ROS levels cause severe damage [46,47]. The rough/glossy phenotype is a result of the lack of cone and pigment cells. The consequence is insufficient secretion of lens material, which directly contributes to the glossy phenotype. This phenotype may occur because cone and pigment precursor cells undergo too few mitotic divisions, failing to enter the second mitotic wave due to insufficient ATP production. Alternatively, the phenotype could result from the misregulation of cone and or pigment cell differentiation. Our results suggest an additional possibility of cell damage and death from ROS after 42h APF. Importantly, antioxidants can partially rescue the glossy eye phenotype in the *dOpa1* intron 3 large clone ommatidia, but do not rescue the rough eye phenotype. This may further implicate the role of ROS in the pathogenesis of the eye phenotypes in the *dOpa1* mutants.

Our results show that loss of *dOpa1* causes a rough, glossy eye phenotype by two distinct pathogenic pathways. The lattice cell death observed in the developing eye is probably linked to increased cytochrome-c release. The glossy eye phenotype in the mutant flies is consistent with decreased lens material secretion from the cone/pigment cells, which are damaged by sustained high ROS levels. Since we were able to partially reverse the eye phenotype through antioxidant treatment and expression of human SOD, antioxidant may provide a fruitful approach for treating this condition.

## Materials and Methods

### *Drosophila* strains and genetics.

The following stocks containing insertions in *CG8479* were used in this study as described in *Results*. y[d2] w[1118] Pi2 P{GMR-lacZ.C(38.1)} TPN1; P{ry[+t7.2]= neoFRT}42D P{w[+mC]=lacW}l(2)s3475[s3475]/CyO y[+] was a generous gift from the UCLA Undergraduate Research Consortium in Functional Genetics (URCFG) [30]. PBac[48]CG8479^f02779^ / CyO y[+] and PBac[48] CG8479^f03594^ stocks were obtained from the Harvard *Drosophila* stock center. The following stocks were used to generate somatic clones. y[d2] w[1118] P{ry[+t7.2]=*ey-FLP*.N}2 P{GMR-lacZ.C(38.1)}TPN1; P{ry[+t7.2]=neoFRT}42D, y[d2] w[1118] P{ry[+t7.2]=*ey-FLP*.N}2 P{GMR-lacZ.C(38.1)}TPN1; P{ry[+t7.2]= neoFRT}42D M(2)w+, and y[d2] w[1118] P{ry[+t7.2]=*ey-FLP*.N}2 P{GMR-lacZ.C(38.1)}TPN1; P{ry[+t7.2]=neoFRT} 42D M(2)w+ Ubi-GFP stocks were also the generous gifts of the UCLA URCFG. The y[1] w[1]; Ki[1] P{ry[+t7.2]=Delta2–3}99B stock was used to generate excisions of P-Element used in this study; it was obtained from the Bloomington *Drosophila* stock center. The following stocks were used to express human superoxide dismutase 1 (hSOD1) in dOPA1^in3^ somatic clones. UAS-hSOD1 transgenic flies were kindly provided by Dr. Bodmer [39], and P{ey3.5-GAL4.Exel} were obtained from the Bloomington *Drosophila* stock center.

### Protein isolation.

30 mg of adult *Drosophila* were suspended in cold PBS and homogenized using a pestle. Cuticles were removed by centrifugation and the remaining tissue lysed in lysis buffer (Aviva), with protease inhibitors (Roche), on ice for 20 minute and used immediately for western blot analysis.

### Western blot analysis of OPA1.

Proteins were separated by 10%–20% Tris-HCL polyacrylamide gel (Bio-Rad) electrophoreses, transferred to PVDF membranes (Invitrogen), probed with a mouse polyclonal anti-OPA1 antibody (1:1,000) (Abnova) and isotype matched secondary antibodies conjugated to horseradish peroxidase (1:10,000). Signals were detected using the ECL Plus reagents (Amersham Biosciences).

### Light and scanning electronic microscopy.

Leica MZ95 dissecting scopes were used and images were acquired with a Qimaging MicroPublisher 5.0. For scanning electronic microscopy (SEM), anesthetized 1–5 days old *Drosophila*, were imaged using a FEI Company, Quanta 600 scanning electron microscope at a resolution of 3.5 nm at 30 kV.

### DNA isolation.

DNA was isolated from single adult *Drosophila* flies by incubating the flies in DNA prep buffer (10mM Tris HCl [pH 8.2], 1mM EDTA [pH 8.0], 25mM NaCl, Proteinase K [200 ug/ml]) for 30 min at 55 °C, followed by inactivation of Proteinase K by 5 min at 95 °C.

### Genotyping.

DNA isolated from *Drosophila* was PCR amplified. Primers flanking the P-element insertion in exon 2 were used to verify the excision of the P-Element. The primer sequences are: F1 5′-GAGATTGAAAGGGCGATGG-3′; R1 5′-CCCACGGACATGCTAAGG-3′. F2 5′-TAAAATTCGGCAGTCCATCC-3′; R2 5′-AATGTGTTTTGCCCACAGG-3′ The PCR products were sequenced, using standard Big Dye 3.1 procedures, in the UCI DNA Core sequencing facility. Primers flanking the junction between the transposon insertion and intron 3 were used to verify the presence of dOPA1^in3^. The primer sequences are: in3F 5′-TCCAGACGACTGTCAAACCA-3′; in3R 5′-CCTCGATATACAGACCGATAAAAC-3′. Primers within the hSOD1 and Gal4 cDNA were used to genotype the presence of the UAS-*hSOD1* and eyeless-*Gal4* transgenes respectively. The primer sequences are: hSOD1F 5′-TGCAGGTCCTCACTTTAATCC-3′; hSOD1R 5′-CTTTGCCCAAGTCATCTGC-3′; Gal4F 5′-TCGATTGACTCGGCAGCTCATCAT-3′, Gal4R 5′-GCGTCTTTGTTCCAGAATGCTGCT-3′.

### Dissection and staining of *Drosophila* retinae.

Pupae were aged at 25 °C. Retinae were dissected into PBS and fixed in 4% Paraformaldehyde/PBS and permeabilized in PBS/0.2% Triton X-100. Primary antibodies were: anti-armadillo N27A1 (1: 10, Developmental Studies Hybridoma Bank, DSHB). Secondary antibodies were Alexa-conjugated (Molecular Probes).

### Confocal microscopy analysis of the *Drosophila* eyes.

Whole anaesthetized *Drosophila* was placed in 35 mm FluoroDish (World Precision Instruments) for imaging with high numerical aperture objectives (40× n.a. 0.8 and 100× n.a.1.0, both water immersion Zeiss Achroplan objectives) on Zeiss LSM 510 META NLO microscope. Eyes were placed in a droplet of water to prevent movement. Ommatidial units were observed using 488-line of argon laser in confocal reflectance and fluorescence (GFP, emission isolated through narrow 500–530 nm bandpass filter) channels simultaneously. Stacks of images were acquired with 0.5 μm or 1 μm steps in Z-direction for 3-D reconstructions. Data were analyzed and processed using the LSM 510 3D software package.

### Transmission electron microscopy.

Whole *Drosophila* were fixed overnight in 4% paraformaldehyde at 4 °C and transferred to a post-fixation solution of 1% glutaraldehyde for at least 1 hour, then rinsed in PBS and placed in 1% osmium tetroxide for 20–60 min, and dehydrated by ethanol and propylene oxide immersion. A flat-embedding procedure was used after which the tissue block was trimmed using a single-edged razor blade under a dissecting microscope (Nikon). A short series of ultrathin (60–80 nm) sections containing ommatidium was cut from each block with an ultramicrotome (Reichert-Jung) and sequential sections were collected on mesh and formvar-coated slot grids. The sections were stained with uranylacetate and lead citrate to enhance contrast. Sections containing granule cells and the hilus were examined with a Philips CM-10 transmission electron microscope and images of ommatidial units were captured with a Gatan digital camera.

### Antioxidant treatment.

Antioxidants were dissolved in solvents according to the manufacturer's (Sigma) protocols and mixed in *Drosophila* media. Solvents were mixed with instant *Drosophila* media as controls [29].

### MitoSOX staining.

MitoSOX staining on the large clone eyes were performed on adult *Drosophila* using a method based on the manufacturer recommendations and as described recently [48]. Briefly, *Drosophila* were dissected swiftly, removing eye tissue containing ommatidial units from the remainder of the head while keeping the eye intact. Eyes were then stained with MitoSOX (2.5mM) in HBBS (Gibco) for 30 minutes at 25°C and then washed 4 times in HBBS and visualized using standard fluorescent microscopy techniques.

## Supporting Information

Figure S1 *Drosophila* CG8479 (*dOpa1)* Is an Ortholog of Human OPA1 An amino acid sequence alignment of human OPA1 (hOPA1), mouse Opa1 (mOpa1), *Drosophila* CG8479 (*dOpa1*), and yeast Mgm1 was performed using MUSCLE (http://phylogenomics.berkeley.edu/cgi-bin/muscle/input_muscle.py). The three major domains of OPA1 and its homologs are highlighted in green (the basic domain), red (the GTPase domain), and yellow (the Dynamin Central Region) (http://lbbma.univ-angers.fr/lbbma.php?id=9). Both National Center for Biotechnology Information (NCBI) BLAST searches and MUSCLE sequence alignments indicate that *CG8479* is a *Drosophila* ortholog of human OPA1.
(4.0 MB JPG)

Figure S2 Crossing Scheme for the Production of and Scoring of Resulting Clone Phenotypes The generation of homozygous somatic mutants with the transposon insertions in both alleles by a series of crosses of the stocks *dOpa1* ^+/in3^ and *dOpa1* ^+/ex14^ (A). The stocks with a transposon insertion in intron 3 (*dOpa1* ^+/in3^) and exon 14 (noncoding region) (*dOpa1* ^+/ex14^) (illustrated by the P and red triangle) were crossed with stocks containing flippase recombination target sequences at cytolocation 42D (FRT42D) (illustrated by FRT42D and the yellow triangle) of Chromosome 2 and flippase under the control of the *eyeless* promoter on the X chromosome, and a white eye yellow body background (yw *ey-FLP*). Males with yellow body and red eyes were selected. F1 progeny contained the expected numbers of all markers. The small clone mosaic males were then crossed to a stock that contained the mutant *Minute* gene (A) to produce large clone. This mutation is homozygous lethal and can be used to select against *dOpa1* ^+/+^ cells. The phenotypes produced by the large clone mosaic eyes of the *dOpa1* ^ex2^, *dOpa1* ^in3^, and *dOpa1* ^ex14^ mutations (*n* > 60) were scored based on the phenotype present (B).
(83 KB JPG)

Figure S3 Homozygous Mutation of *dOpa1* Results in Necrosis of Cells in the Large Somatic Clones of the Adult *Drosophila* Eye Bright field microscopy images of the adult eyes of large clones of the *dOpa1* ^ex2^ and *dOpa1* ^in3^ are shown in (A) and (B), respectively. The onset and penetrance of this phenotype was variable, but was observed many times throughout the study.
(108 KB JPG)

Figure S4 *Drosophila* Homozygous for the Excision of *dOpa1^ex2^* Are Homozygous Viable and Do Not Exhibit Any Abnormal Phenotypes The crossing scheme for the excision of the P-element from exon 2 is illustrated in (A). (A) *Drosophila* homozygous for the excision reversing the mutation is illustrated in (B) (eye) and (C) (body).
(452 KB JPG)

Figure S5 Homozygous Mutation of *dOpa1* Results in Severe Disorganization of Ommatidial Units as a Result of Missing Interommatidial Cells Pupae staged 42 h after pupae formation (APF) and mosaics with GFP expression in the eye imaginal disk were collected and stained with Hoechst and for Armadillo. A fluorescence micrograph of ommatidial units containing GFP negative (*dOpa1* ^in3/in3^ *Minute* ^+/+^ *ubi-GFP* ^−/−^ clones) and GFP positive ommatidial units (*dOpa1* ^+/in3^ *Minute* ^+/−^ *ubi-GFP* ^−/+^ clones) is illustrated in (A). The white box marked with a d represents the area illustrated in Figure 4A in more detail. (B) illustrates *dOpa1* ^in3/in3^ *Minute* ^+/+^ ubi*-GFP* ^−/−^ ommatidial units outlined in white. Note the severe disorganization of the *dOpa1* ^in3/in3^ *Minute* ^+/+^ *ubi-GFP* ^−/−^ommatidial units compared to the *dOpa1* ^+/in3^ *Minute* ^+/−^ *ubi-GFP* ^+/−^ ommatidial units. *dOpa1* ^+/in3^ *Minute* ^+/−^ *ubi-GFP* ^+/−^ ommatidial units and *dOpa1* ^in3/in3^ *Minute* ^+/+^ *ubi-GFP* ^−/−^ ommatidial units were scored for the number of different cell types based on morphology (C).
(376 KB JPG)

Figure S6 Crossing Scheme for the Production *dOPA1* Mutant Large Clone Phenotypes with *UAS-hSOD1* and *ey-Gal4*
The stock with a P-element insertion in intron 3 (*dOpa1* ^+/in3^) (illustrated by the P and red triangle) and FRT42D and yw *ey-FLP* on the X chromosome were crossed with stocks containing *UAS-hSOD1* transgene on Chromosome 2 [39] (A). Males without the CyO balancer were selected and crossed to new females of the same genotype as in P. Females without CyO balancer were then selected and crossed to males with yw *ey-FLP* on the X chromosome and FRT42D on the second chromosome. Mosaic-eyed male progeny were then crossed to a stock that contained the CyO balancer to establish stocks. The genomic DNA of F3 males was isolated and genotyped for the presence of the *hSOD1* transgene and transposon insertion in intron 3. Established stocks were genotyped after they had been established. The production of dOPA1 large clones with both UAS*-hSOD1* and *ey-Gal4* were established by the crossing scheme illustrated in (B). Stocks containing yw *ey-FLP* on the X chromosome as well as FRT42D, the dOPA1^in3^ transposon insertion, and UAS-*hSOD1* were crossed to stocks with the *Gal4* transgene under the expression of the *eyeless* promoter (*ey-Gal4*) on the third chromosome. Straight-winged males were then crossed to a stock that contained the mutant *Minute* gene to produce large clones. Large clones were scored and genotyped for the presence of the *Gal4* transgene.
(566 KB TIF)

Video S1 *dOPA1* Large Clone Blue (221 KB MPG)

Video S2 *dOPA1* Large Clone Green (39 KB MPG)

Video S3 *dOPA1* Large Clone Reconstruction (251 KB MPG)

## Abbreviations

DOA: autosomal dominant optic atrophy
IOC: interommatidial cell
OPA1: optic atrophy gene 1
ROS: reactive oxygen species
